# Effectiveness of Health Promotion Interventions in Primary Schools—A Mixed Methods Literature Review

**DOI:** 10.3390/healthcare11131817

**Published:** 2023-06-21

**Authors:** Joca Zurc, Camilla Laaksonen

**Affiliations:** 1 Department of Pedagogy, Faculty of Arts, University of Maribor, 2000 Maribor, Slovenia; 2 Faculty of Health and Well-Being, Turku University of Applied Sciences, 20520 Turku, Finland

**Keywords:** school health, health promotion, intervention effectiveness, mixed methods review

## Abstract

School-based health promotion interventions (HPIs) are commonly used in schools, but scientific evidence about the structures of effective interventions is lacking. Therefore, we conducted a mixed methods systematic literature review to recognize the HPI structures related to their effectiveness. Based on the inclusion criteria, 49 articles were selected for the literature review. The articles, published in 2011–2022, described 46 different school-based interventions conducted in 20 different countries. The average duration of the interventions was 12 months, and they were implemented mostly with an RCT study design (61.2%) and by targeting children (69.4%). Three main groups of interventions were identified and explained: (1) extensive and long-term interventions; (2) school policy-changing interventions; and (3) highly effective interventions. Effective school-based HPIs included multiple target groups, multiple providers with external experts, and an efficient duration and timing of follow-ups. The implications for educational research and school practice are presented. Evidence on the effectiveness of health-related interventions is still lacking and needs to be addressed in further studies.

## 1. Introduction

Children of 6–12 years of age experience good health in general. However, at the same time, unhealthy behaviors, such as a sedentary lifestyle, unhealthy nutrition, obesity, use of substances, and lack of hygiene, are fairly common. School-aged children also frequently report suffering from psychosomatic symptoms, poor mental health, and growing health inequality within and between countries [1,2,3].

Hence, school-based health promotion interventions (HPIs) are widely used to promote school children’s physical and mental health. Well-implemented HPIs can improve children’s health, academic achievements, and completion rate, reducing risk factors and contributing to the bridging of health inequalities [4,5,6,7]. Positive outcomes of those interventions were also found for the underachieving and under-represented groups of school children [8,9]. On the contrary, limited scientific knowledge exists on the effectiveness of these interventions [10,11]. Rare cases of previous systematic reviews focused merely on the evaluation of interventions in specific areas of health, such as obesity-related outcomes [12], physical activity [13], dietary behavior [14], or postural health [15]. However, assessing the effectiveness of implemented HPIs is particularly important to understand why the intervention works or does not work and to distinguish between the components of the intervention structure that affect its outcome.

Considering the lack of comprehensive analyses of the effects of HPIs in primary schools in a broader context, we design the mixed methods literature review aiming to determine (1) what types of school-based HPIs have been reported, (2) what is the effectiveness of the reported school-based HPIs, and (3) what are the main elements or components of school-based HPIs significantly related to their effectiveness. This literature review focuses on the broad international perspective by including all reachable empirical studies on the effects of HPIs in primary schools with no restriction on the country of origin, school status (private/public), or participants’ nationality, financial status, and gender.

### 1.1. Promoting Health in Schools

Primary schools are central communities for children as more than 90% of children aged 5–15 are enrolled in primary schools globally. School-based interventions reach practically all diverse groups of children and parents [16,17]. Teachers, school nurses, and other school professionals are well positioned to respond to the need for health promotion, illness prevention, and early intervention related to a child’s health and academic success [18]. 

Schools have been reported to provide ideal settings for regulations that reduce the risk of noncommunicable diseases, promote a healthy lifestyle, and prevent unhealthy behavior habits [16,19,20,21]. Moreover, research findings imply the importance of fostering social–emotional well-being and social–emotional competencies (e.g., self-knowledge, self-esteem, self-regulating emotions, assertive communication skills) in preventing behavioral problems (e.g., social deviance, bullying, disruptive and aggressive behavior), problematic Internet use, and school dropout among primary school children and adolescents [22,23]. Namely, effective psycho-educational interventions for promoting well-being in schools were found to be related to improving behavioral outcomes and socio-emotional skills, higher academic achievements and completion rates, and an overall positive school climate [22,24,25]. 

### 1.2. Components of the School-Based HPIs Related to the Effectiveness

Studies reveal diverging findings on the effectiveness of school-based HPIs, recognized as interventions with positive effects, partial effects, and potentially unintended reverse effects [11,12,26]. The success of the school-based HPIs depends on different factors, including structural elements of the intervention. Previous empirical and review studies revealed a relatively broad range of HPI structural elements that might affect the effectiveness and implementation quality of the HPIs and potentially impact children’s health, such as the following:

- A program for the intervention (e.g., area of health, social–emotional learning and behavioral skills) [11,27];
- Participants or target groups (e.g., children, parents) [11,28];
- Providers (e.g., teachers, external experts, interdisciplinary teams) and training for them [10,11,27,29];
- School contextual factors (e.g., policy, organization capacity, collaboration with local communities and stakeholders) [10,11,28,29].

Multicomponent interventions, including policy change, parent involvement, cooperation between educational and other experts, training for providers, and working with local communities, seem more effective than single-component HPIs [10,27,28,29,30]. However, any intervention effects may be relatively limited or average due to a lack of self-reported data on long-term follow-up effects [30].

The presented systematic overview frames possible factors that may impact the effectiveness of the school-based HPIs. Therefore, it should be studied more systematically and comprehensively to understand which structural elements or components of the HPIs contribute to the highly effective HPIs in primary schools and significantly impact children’s health and lifestyle.

## 2. Methods

**Figure 1:**
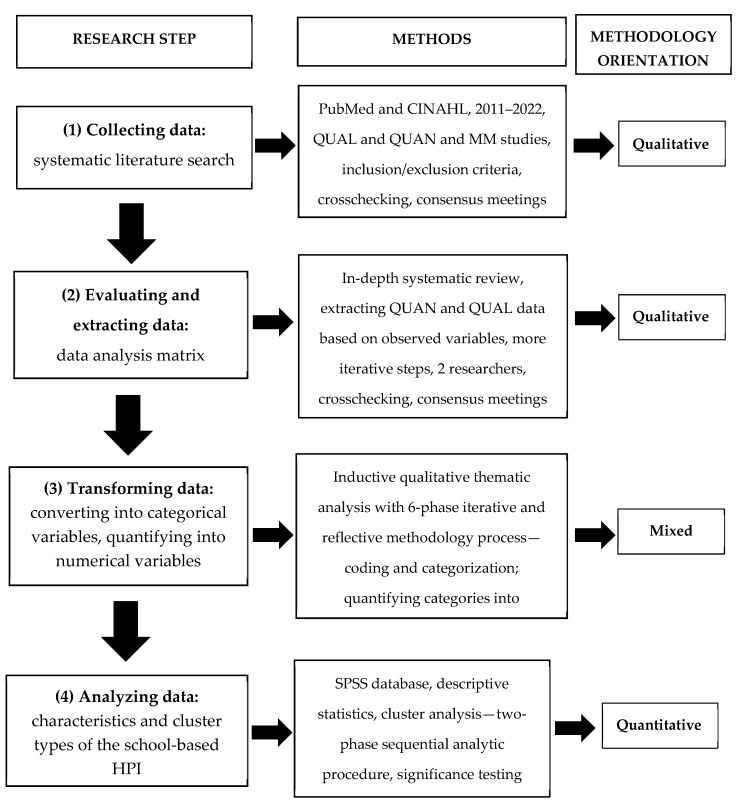
Research design of the mixed methods systematic literature review.

The mixed methods approach with quantitative and qualitative methods was used to obtain a more extensive, complex, and in-depth insight into the effectiveness of HPIs in primary schools. This study followed the detailed protocol that has been created in guidelines for mixed methods literature reviews [31,32,33] (see detailed description in Section 2.1). It was conducted in four research steps: (1) collecting data—HPI evidence published in peer journals; (2) evaluating and extracting data according to observed variables; (3) transforming data into categorical or numerical variables; and (4) analyzing data. Different methodological orientations were used in this literature review, such as qualitative-driven collecting, evaluating, and extracting data, mixed-driven data conversion, and quantitatively driven data analysis (Figure 1). 

### 2.1. Conducting the Mixed Methods Literature Review

A mixed methods literature review “refers to any combination of methods where one significant component is a literature review (usually systematic)” [31] (p. 94). This approach combines qualitative and quantitative components within the same study [34]. The convergent parallel mixed methods design was carried out in this study, enabling us to conduct qualitative and quantitative parts with the same priority [35]. 

In the literature, we found three ways in which the reviews could be mixed [32]: (1) the types of studies included in the review are quantitative, qualitative, and mixed; (2) the synthesis methods used in the review are mixed (e.g., systematic review with meta-analysis and meta-synthesis); (3) two analytical approaches are incorporated in the review: theory testing (quantitative) and theory building (qualitative). According to the first type, this review was not limited to any methodological orientation; quantitative, qualitative, and mixed studies were welcome in this analysis. However, since the field of HPI evaluation is more quantitatively orientated (e.g., randomized controlled trial design), quantitative studies dominated the literature search and selection. This review focuses on mixing qualitative and quantitative approaches in data analysis and synthesis according to the second type of mixed methods review, as defined above [32]. 

Furthermore, the mixed analysis strategy was carried out for merging, transforming, and comparing the two separate data stands [32,33]. After extracting quantitative and qualitative data from the selected articles separately, some qualitative narrative data were quantified into numerical variables (e.g., a 5-point scale to evaluate HPI effectiveness) or converted into categorical variables (e.g., categories of intervention providers) for further statistical analysis. On the other hand, quantitative data (e.g., sample size, intervention duration) were converted into categorical variables for further qualitative analysis. A detailed description of this transforming analysis is described in Section 2.3.

### 2.2. Procedure of the Literature Search and Literature Selection

**Figure 2:**
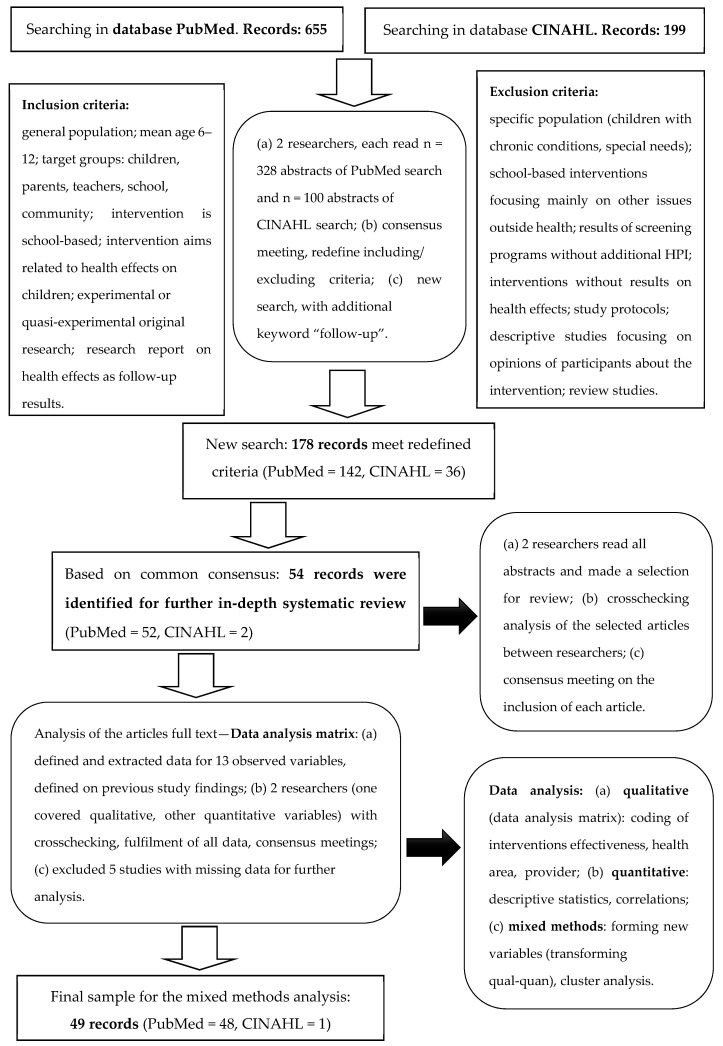
Flow diagram of the literature search, selection, and data analysis strategy.

First, the search strategy and literature selection were carried out on principles of systematic literature review. With this approach, we systematically searched, appraised, and synthesized research evidence on school-based HPIs. The comprehensive search until 11 October 2022 was carried out in two databases, PubMed and CINAHL. These two databases were selected purposively as we were particularly interested in medical, biomedical, and public health randomized controlled trials (RCTs) of HPIs, which are well represented in the PubMed database. Additionally, our study focused on school-based HPIs, which are, in many countries, delivered by the school nurse. Therefore, the comprehensive nursing science database of CINAHL was included. Scopus, Web of Science (WoS), and other databases were not used due to restrictions of availability for authors’ institutions and particular fields of interest being most precisely and comprehensively represented by literature in PubMed and CINAHL databases. The search keywords were “school-based” AND “intervention” AND “follow-up”. The search was limited by activating the following filters: journal research article with abstract, published from 11 October 2011, and study population based on children between 6 and 12 years of age. Figure 2 represents the search strategy with precisely determined inclusion and exclusion criteria and the data analysis procedures. 

The first PubMed search resulted in *n* = 655 hits, and the CINAHL search resulted in *n* = 199 (*n* = 854). After redefining the including and excluding criteria in a consensus meeting between two researchers and an additional search with the keyword “follow-up”, the new search for school-based HPIs resulted in *n* = 178 records. Both researchers read all abstracts independently to ensure the validity of the inclusion/exclusion of articles based on the stated inclusion and exclusion criteria in Figure 2. After excluding duplicates and reading the abstracts, we selected *n* = 54 full-text articles for an in-depth systematic review.

### 2.3. Data Extraction and Categorization

In the first stage, the comparable data matrix was designed and used for data extraction from the selected articles. All (*n* = 54) full-text articles were read, and data were extracted by observing 13 different variables. This process was based on qualitative research methods. The observing variables were determined based on preliminary scanning of selected articles using deductive and indicative approaches. The comparable data matrix included general information for each study under review, such as author and country of intervention implementation. Further, intervention features such as the type of intervention (study design), population and sample, age group, intervention duration, number of follow-ups, follow-up outcomes, target group, intervention provider, health area of intervention, and intervention effectiveness were extracted. More iterative steps were conducted in data extraction from the selected *n* = 54 articles and designing a data matrix. Two researchers with health promotion, education, and methodology backgrounds independently summarized the data from full-text articles and crosschecked their solutions.

In the second stage, after data extraction was completed, inductive qualitative thematic analysis with a six-phase iterative and reflective methodology process [36] was conducted. In this process, the extracted qualitative data, such as the health area of intervention, research design, target groups, and intervention providers, were analyzed by the qualitative inductive approach of generating initial codes and, afterward, merging codes into categories and defining themes with common features. As a result of this process, new measurable items were introduced and used in further analysis. 

First, the intervention effectiveness was qualitatively analyzed by thematic analysis using a deductive approach. A theoretical concept on the principles of HPI evaluation [37] was applied to determine three broad categories of effectiveness for every intervention under study: (1) reverse or no effect (lower results than at baseline), (2) partial effect, and (3) positive effect. In the next step, the defined categories were transformed into a quantitative variable with a 5-point interval scale (1, reverse effect; 2, no effect; 3, partial/moderate effect; 4, positive effect; 5, strong positive effect). The effectiveness of interventions was determined based on the reported findings at the intervention completion. A strong positive effect (4) was assigned to the HPI, showing a positive effect that remained at follow-up(s). This transformation process was based on the mixed methods quantification approach [35] and resulted in a new variable used in statistical analysis. 

The identification of codes and themes was conducted by one researcher and crosschecked by the other researcher. A final decision was made with a consensus based on the differences discussed between the researchers in more consensus meetings until an agreement was reached. The results of the qualitative thematic analysis and data transformation are presented in Table 1.

The extracted quantitative data (sample size at baseline, intervention duration in months, frequency and time of follow-ups after intervention cessation) and age of participants (mapping with school grade system in the country of HPI origin) were also classified into categorical groups. However, the raw numerical data from the reviewed studies were applied in the statistical data analysis (Section 2.4).

Once the extracted data matrix was completed, an electronic database was designed in the SPSS 26.0 software package (SPSS, Chicago, IL, USA). In this process, five additional articles were excluded as they failed to report complete data on extracting variables (e.g., sample size, intervention duration). The SPSS database was completed for 49 reviewed articles that entirely met the inclusion criteria and resulted in the 23 variables extracted directly from the literature or transformed. The created SPSS database was used for further statistical analysis based on descriptive statistics and multivariate cluster analysis.

### 2.4. Quantitative Data Analysis

A cluster analysis was implemented to identify different types of school-based HPIs with common features related to their effectiveness. Cluster analysis was highlighted as a highly appreciated approach for detecting patterns of health-related behaviors [38]. Segregation of the observed variables enables identifying well-established and at-risk groups. “This process allowed identifying the number of clusters that maximizes differences between clusters or groups and minimizes within-group differences on the dependent variables” [39] (p. 92). The final goal is to organize large quantities of multivariate information by forming homogeneous groups from the heterogeneous sample. We employed two different multivariate cluster analyses with a two-phase sequential analytic procedure [39]. First, we implemented a hierarchical cluster analysis with Ward’s method; afterward, we implemented a non-hierarchical K-means cluster analysis. In both analyses, a minimized square Euclidean distance was used as a criterion of the differentiation between pairs of units, which represents a measure of similarities between pairs of units (internal cohesiveness) and differences between the groups of units (external insulation) [40]. 

The hierarchical and non-hierarchical cluster analysis included 49 studies and 10 dependent variables, such as intervention type/design of the study, sample size at intervention baseline, the average age of participants at intervention baseline, intervention duration (months), number of follow-ups after intervention cessation, the time of the first follow-up (months after intervention cessation), the time of the last follow-up (months after intervention cessation), number of intervention providers, number of target groups, intervention effectiveness (5-point scale). Before the cluster analysis was employed, all data were standardized, and the measured scores were transformed into standardized z-scores (M = 0, SD = 1) [39,40]. This process was necessary for the comparison between different measurement scales. 

The findings of both applied cluster analyses were compared, and the best solution was taken for the final classification of the HPI clusters. The obtained clusters were labeled with descriptive names based on common features identified by descriptive statistics, one-way analysis of variance, and chi-square test of all 23 studied variables defining the structure of the school-based HPIs. The *p*-value of ≤0.05 was considered statistically significant. The statistical analyses were conducted using SPSS 26.0 software (IBM SPSS, Chicago, IL, USA).

## 3. Results

The review included *n* = 49 articles that described *n* = 46 different school-based HPIs. Only three of the reviewed interventions, namely “Kids N Fitness” [41,42], “Fit-4-Fun program” [43,44], and “KISS” [45,46], were represented by the two publications, and both of them were included in the analysis. All other interventions were selected as individual publications. The results revealed the characteristics of the reviewed HPIs and their effectiveness and cluster types.

### 3.1. Characteristics of the School-Based HPIs

Table 2 presents the main descriptive features of the reviewed interventions, which have been conducted in 20 different countries. Most of them were implemented in Europe (*n* = 27, 55.1%), dominantly based on the RCT research design (61.2%), and targeting 18 different areas of health, mainly addressing dimensions of physical health (*n* = 40, 81.6% studies), such as physical activity (*n* = 8, 16.3%), a balanced diet (*n* = 7, 14.3%), obesity (*n* = 6, 12.2%), and infection (*n* = 6, 12.2%). Less attention was given to mental health interventions (*n* = 9, 18.4%). Stress management, mental disorders, sleep quality, body image, and substance abuse prevention were addressed in these cases. Most interventions (73.5%) were implemented for up to one year and included up to 1200 children (71.4%). The number of follow-ups after intervention cessation ranged between one and six follow-ups. However, more than half of the studies (57.2%) reported only one follow-up, and a quarter (26.5%) reported two follow-ups up to 12 months after the intervention cessation. The analyzed HPIs were mainly targeted at children (*n* = 34, 69.4%) and provided by regular school staff by changing the established school policy or curriculum (*n* = 23, 46.9%). Nevertheless, more than a third of interventions (*n* = 17, 34.7%) were delivered by specially qualified teachers who completed a training program or by external experts (e.g., physiotherapist, school nurse, registered nurse, physician, dietitian, kinesiologist, psychologist, behavior therapist, mental health specialists). Furthermore, some interventions were implemented with more than one provider (*n* = 9, 18.4%).

### 3.2. Effectiveness of the Interventions

**Figure 3:**
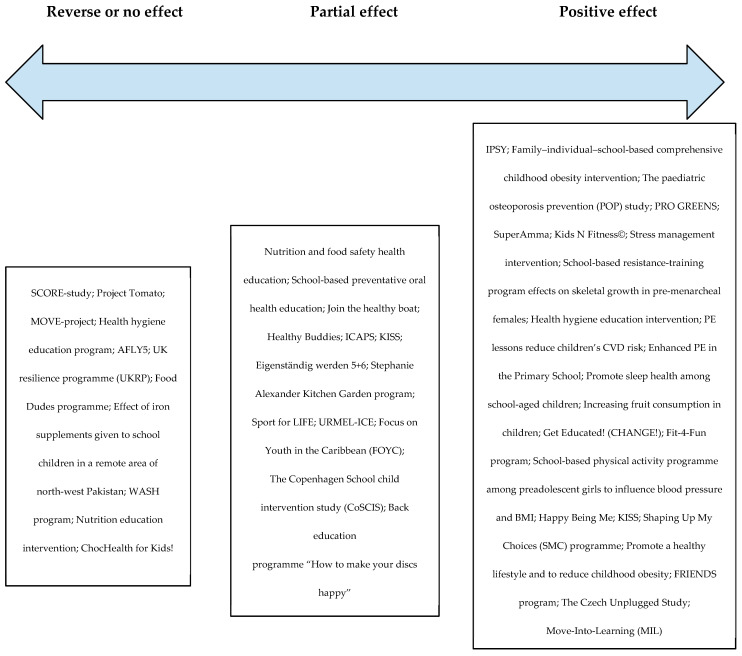
Effectiveness of the school-based HPIs included in the review: HPIs with reverse or no effect [47,48,49,50,51,52,53,54,55,56,57], HPIs with partial effect [46,58,59,60,61,62,63,64,65,66,67,68,69] and HPIs with positive effect [41,42,43,44,45,70,71,72,73,74,75,76,77,78,79,80,81,82,83,84,85,86,87,88,89].

On average, school-based HPIs reported positive effects (Table 3, Mean = 3.39, SD = 1.08). Figure 3 categorizes the analyzed 49 interventions into three groups according to their effectiveness. Half of the HPIs were classified in the group of interventions with a positive effect (*n* = 25, 51.0%), followed by the group of interventions with a partial effect (*n* = 13, 26.5%), and the group of interventions reporting no or reversed effects (*n* = 11, 22.5%). A strong positive outcome, which resulted in a remaining positive effect of intervention up to the last follow-up measure, was reported in seven interventions (14.3%). However, it is essential to highlight two interventions that reported a reverse effect, where the condition of participants even worsened after the intervention compared to the baseline [47,48]. 

### 3.3. Cluster Types of the School-Based HPIs

**Figure 4:**
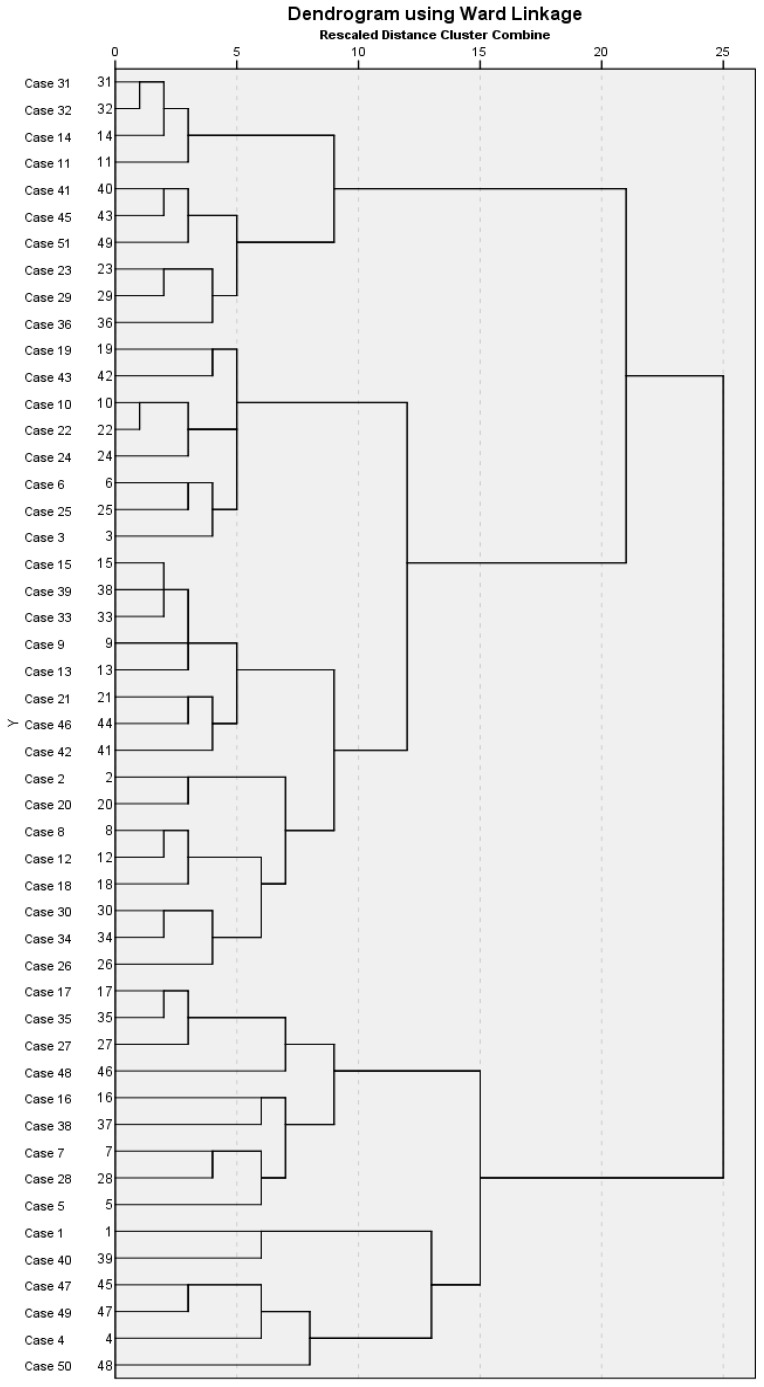
Dendrogram of hierarchical cluster analysis for the school-based HPIs.

We used the multivariate cluster analysis approach to obtain deeper insights into the structure of the studied school-based HPIs related to their effectiveness. First, hierarchical cluster analysis with dendrogram classified all 49 studied interventions that meet the criteria into three cluster groups (Figure 4). Second, a non-hierarchical K-means cluster analysis compared the solution from the first cluster analysis. K-means extracted three groups of school-based HPIs with similar distributions of cluster centers and mean scores on clustering variables, thus confirming the initial solution of the hierarchical cluster classification as the final one. 

In the first cluster group, 15 interventions (30.6%) were classified, the second cluster group consisted of 24 interventions (49.0%), and the third group cluster consisted of 10 interventions (20.4%). All three revealed cluster groups differ in interventions’ effectiveness (F = 5.519; *p* = 0.007), and seven observed structural components showed statistically significant differences. The three types of school-based HPIs represent homogeneity between the interventions within the same group. At the same time, each group significantly differed from the other groups in the following structural elements: effectiveness, duration, follow-ups (number of times, time-frame of the first and the last follow-up), providers, and target groups, as presented in Table 3.

#### 3.3.1. Cluster 1: “Extensive and Long-Term HPI”

The most extensive interventions with a long-term duration were classified in cluster group 1. In 73.3% of cases, these interventions were based on the RCT design. The average sample of participants at baseline was 1.802, two times more than the average sample in cluster 2 (M = 902) and almost three times more than that in cluster 3 (M = 635). Consequently, the duration of the interventions was the longest (more than 25 months), with the highest number of follow-ups (two or three on average). The first follow-up was implemented on average within a year and a half, and the last follow-up was more than three years after the HPI cessation. Moderate effectiveness was found for the school-based HPIs in cluster 1, which consisted of seven interventions (46.6%) with a positive or strong positive effect and five interventions (33.3%) with a partial effect. In addition, in this group, two interventions were classified with a reverse or negative effect on children’s health [47,48].

#### 3.3.2. Cluster 2: “School Policy-Changing HPI”

A specific feature of cluster group 2 was found in a target group intended to reach via intervention. Namely, these interventions targeted merely children, while the other clusters targeted, besides children, their parents, peers, teachers, other school professionals, etc. Furthermore, HPI providers in group 2 were in half of the cases (56.5%) schools with regular curriculum or policy changes. Interventions in group 2 were defined with moderate effectiveness but slightly lower than in group 1, sorted into the three approximately equal subgroups that reported a positive effect, partial effect, and no effect on children’s health.

#### 3.3.3. Cluster 3: “Highly Effective HPI”

The highest intervention effectiveness was revealed in cluster 3. Ninety percent of all HPI classified in this group reported positive effects on children’s health. Furthermore, four studies in this group [43,44,87,89] demonstrated strong positive effects. Only one study [64] was categorized as a partially positive effective intervention, and none showed no effect or reverse effect. 

The cluster analysis showed no statistically significant differences between the three revealed groups in the health area of intervention. In all three groups, physical health was dominantly addressed. Additionally, no significant differences were found in the age of participants, research design, sample size, and region of the intervention origin.

### 3.4. The Relationships between Structures of HPIs and Their Effectiveness on the Mental and Physical Well-Being of Primary School Children

The question of what makes the HPIs effective in enhancing children’s health arises. Which are the common structural elements of the HPIs, and how are they characterized to distinguish the highly effective HPIs from the others? Table 3 integrates and summarizes qualitative thematic and statistical analysis findings in presenting several crucial structural features of effective school-based HPIs.

First, the interventions in the most effective cluster 3 targeted at least one other group of participants, e.g., parents or the whole family, teachers, school management or other staff, peers, or people in the community, besides children. Thus, it could be confirmed with findings in cluster 2, when less effective interventions targeted merely children.

Second, effective school-based HPIs were mainly delivered by multiple providers, usually defined as a multidisciplinary team of professionals or experts in education, healthcare, social care, administration, school management, or evaluation. Providing a school-based HPI in the cooperation between a school and other health-related settings, such as a community with healthcare resources, again provided successful outcomes in infection control [74] and obesity prevention [41,86]. Moreover, interventions supplied by teachers specially trained to implement the intervention activities as part of their usual classroom curricula were highly effective [75,81,82]. Additionally, the active participation of an external expert in program implementation would make the school-based HPI highly promising. According to this literature review, even graduate students [87] or researchers [77] as external providers seem to contribute significantly to school-based HPI effectiveness on children’s health.

Third, the structural elements of an effective HPI could also be characterized by quantitative variables, such as the sample size of participants at the baseline, the duration of the intervention program in months, the number of follow-ups, and their timing after the intervention cessation. While the sample size was not recognized as a distinguishing factor among the revealed cluster groups, on the contrary, the intervention duration with follow-ups had significant impacts. Interventions classified in the cluster of “highly effective HPI” had the shortest time in duration (on average 4.5 months) as compared to the cluster of “school policy-changing HPI”, with three months on average longer interventions, and particularly to the cluster of “extensive and long-term HPI” with interventions for more than two years on average. Interventions with longer duration embedded more follow-ups with an enormous time distance from the intervention cessation, particularly visible in the first cluster group of “extensive and long-term HPI”. On the contrary, the second cluster group of “school policy-changing HPI”, showing the lowest effectiveness, demonstrated the lowest number of follow-ups irrespective of the HPI duration. Consequently, the effectiveness of these interventions on children’s health was very low [51,55].

Ultimately, findings showed that the effectiveness of the HPIs depends on the different structural components of their implementation. Interactivity between quantitative factors (e.g., sample size, duration, follow-ups) and qualitative factors (e.g., quality of research design, interventional program and activities, the expertise of providers, and target groups) seems to play a crucial role in the quality of HPI implementation and effectiveness on children’s health.

## 4. Discussion

Based on the innovative mixed methods literature review with the integration of quantitative and qualitative data collections and analysis, supported by a systematic review, this study aimed to identify structures of the school-based HPIs and their features related to effectiveness. To the best knowledge of the authors of this paper, this is the first study to use a mixed methods literature review with multiple cluster analysis, primarily targeting a literature review of the school-based HPIs. The findings showed a wide variety of school-based HPI implementations, mainly related to their research protocol (RCT), sample sizes of participants, targeted groups, intervention providers, duration, follow-ups, and targeted health area. Among them, target groups, providers, and duration of follow-ups seem to be the strongest predictors of school-based HPI effectiveness. 

### 4.1. Cluster Types of the School-Based HPIs

The main three groups representing the three different types of school-based HPIs were revealed by multiple cluster analysis. The HPI effectiveness, target groups, intervention providers, period of duration, and follow-ups after the intervention resulted in the most considerable differentiation among them and the most significant homogeneity within them. The most effective school-based HPIs were revealed in cluster 3, “highly effective HPI”, where 90% of all HPIs classified in this type reported positive effects on children’s health. Moderate effectiveness was identified for interventions in cluster 1, “extensive and long-term HPI”, and cluster 2, “school policy-changing HPI”. However, the school-based HPIs reported a positive or strong positive effect that was slightly more prevalent in cluster 1.

Interestingly, we found that most interventions included in this review targeted only children, and half of them were provided by the regular school curriculum. However, there were some differences between the three cluster types. In particular, interventions in cluster 2 targeted only children and were in half of the cases provided by the regular school curriculum/policy changes. In contrast, besides children, the HPIs in the other two clusters targeted their parents, peers, teachers, and other school professionals. Multiple target groups and multiple providers, represented by specially trained schoolteachers and external experts, were mainly characteristic of highly effective HPIs in cluster 3.

The most extensive interventions, defined by a long-term duration, more follow-ups, and the most distant times of the first and last follow-ups after the intervention cessation, were classified in cluster 1. In most cases, these interventions were based on the RCT design. Similarly, cluster 2 consisted of a substantial part of the RCT interventions. Surprisingly, both cluster types were characterized by moderate effectiveness, which indicates the importance of avoiding judging the effectiveness of the HPIs solely by considering the research design. Implementing the RCT design should not be taken for granted as high-quality; on the contrary, a critical evaluation of the HPI quality of implementation and its effects on children’s health should be carefully considered.

Finally, this study found homogeneity among the three cluster types of school-based HPIs in predominantly addressing the physical health, age, and sample size of children who participated in interventions. The similarities and differences identified in the three cluster types of school-based HPIs in our study contribute to the understanding of structural elements related to the interventions’ effectiveness and their potential impact on children’s health and lifestyle. These findings align with previous research indicating that multicomponent interventions seem more effective than single-component HPIs [27,28,29,30] and arguing that the lack of evidence of effective school-based HPIs is due to unsystematic and inadequate scientific research [11,12,30,90,91].

### 4.2. Key Structural Elements of the Effective School-Based HPIs

Both this review and previous studies suggested that interventions aimed at promoting health in child populations should include multiple providers and multiple target groups through the engagement of schools, health providers, and families [27,28,29,92]. These interventions resulted in several long-lasting benefits for children, such as higher academic achievements [7,80], more active lifestyle [43,44], obesity prevention [62,86], and increased social–emotional well-being and competencies [24,25]. The inclusion of multiple providers, specially trained teachers, and external experts, targeting different groups besides children, such as family members, peers, teachers, and the wider community, was demonstrated in this study as a strong predictor of HPI effects on children’s health. For example, a powerful family–individual–school-based comprehensive intervention model for controlling childhood obesity was outlined [71].

According to previous research, most school children report good physical health, but challenges related to health’s emotional and social dimensions remain [1,2]. Similarly, in this review, most school-based HPIs targeted physical health dimensions such as cardiovascular disease prevention, promotion of healthy lifestyle and oral health, injury prevention, and infection control. Thus, it initiates a discussion about the justifiability of mental health-related interventions, their potential benefits for empowering child’s mental health, and managing the pressures of academic achievements [6].

Following the findings of this review, it seems that even less extensive HPIs, delivered by the class teacher, in a couple of months, within a regular curriculum with wisely selected intervention activities, including follow-ups, teachers’ training, external collaborators, and more target groups, have a long-lasting and sustainable impact on children’s health. Conversely, somebody would predict that longer interventions (e.g., longitudinal studies) are more likely to be successful because of better resources, support, training, and information delivered to participants over an extended time. However, a highly effective group of HPIs in our study demonstrated the contrary. Hence, a school-based HPI with undemanding time implementation could also be influential. For example, only a 10 min daily intervention on stress management performed for four months by schoolteachers was reported to have a positive effect on anxiety symptoms and heart rate variability at the 1-year follow-up among 8-year-old children [75]. Moreover, a strong positive impact of the school-based HPIs was reported even within shorter interventions, for instance, up to two months, on the level of physical activity and better health-related fitness [42,43,44]. Moreover, under-represented and delayed follow-up(s) may decrease the effectiveness of the intervention. For example, a 5-year RCT on infection control with only one follow-up, 12 months after intervention cessation, showed higher intensity of infection among children compared to the baseline [47]. 

Ultimately, based on this review, effective school-based interventions are commonly reported around the globe and focus most commonly on physical health. The effectiveness of interventions depends on different structural components of implementation. Interactivity between quantitative factors (e.g., sample size, duration, follow-ups) and qualitative factors (e.g., quality of research design, interventional program and activities, the expertise of providers, and target groups) seems to play a crucial role in the quality of HPI implementation and effectiveness on children’s health. 

### 4.3. Implications for Educational Research and School Practice

These review findings emphasized that teachers, school nurses, and other health and education professionals are critically positioned to develop evidence-based HPIs to promote healthy development and progressive academic achievements for primary school children. Their success depends on how they are educated and skilled to design, conduct and follow HPIs in a school setting. This research suggests several recommendations for schools.

First, designing a high-quality HPI based on the best knowledge and including effective structural components recognized in our review is highly recommended. Second, according to this review, interventions in primary schools seem most commonly to target the physical health dimensions. Hence, further school-based interventions to equally address children’s emotional and social dimensions of health are recommended. Third, researchers and other healthcare and pedagogy professionals should participate in designing, implementing, and following the interventions to add a solid knowledge of the safety, effects, and ethical dimensions of school-based HPIs. Fourth, the support of school decision-makers to ensure the availability of resources, such as staff and training for them and resources for implementing HPIs at schools, is strongly encouraged.

### 4.4. Limitations of the Present Review and Directions for Future Studies

This review has some limitations that must be addressed and considered when interpreting its findings. First, the literature search for the study was performed only in two databases (PubMed and CINAHL) with some additional search limiters. These factors may be reflected in the retrieved and analyzed data. PubMed and CINAHL are, however, commonly available and used databases for researchers in the field of medicine and health sciences, which may limit the review from interventional studies in other fields, such as education, psychology, and kinesiology. 

Second, as the mixed methods literature review was a challenging and time-consuming task, 21 months passed between the literature search and the writing of the first draft of this article. Therefore, it can be expected that some relevant studies on school-based HPI effectiveness were not included. However, we strongly believe that conclusions based on the systematic and comprehensive review of 49 school-based HPIs with in-depth mixed methods data analysis brought new knowledge and understanding to the field. 

Third, according to this and previous studies, it must be emphasized that descriptions of the school-based HPI structural elements highly varied between studies. Remarkably, studies inconsistently reported the dropout rate of sample size during intervention and follow-ups. Similarly, the lack of description of follow-up measurements, without explaining activities between the intervention cessation and the last follow-up, was also notable in the reviewed articles. All borderline cases were discussed between the researchers, and the final decision was made with a common consensus. However, a strong need for additional rigorous systematic meta-analysis calls for future reviews. 

Fourth, this review solely analyzed the HPI structural elements without focusing on the content of the intervention activities and material for participants. This perspective plays a crucial role in the HPI effectiveness and, therefore, should be carefully examined in future studies from different aspects of analysis, e.g., a didactical view, HPI sensibility for participants of diverse backgrounds such as socio-economic status, school district/region, race, gender, or personal characteristics related to health. Further research is needed to understand the impact of interventions on health equality. Mandatory primary school education offers a unique opportunity to reduce those inequalities and encourages better health for all children and their families, no matter their circumstances.

Fifth, the highly effective HPIs were considered in the spotlight in this review. Future studies should also look more carefully at the interventions with small success or even reverse effects. In this context, particularly ethical issues on the reverse and unintended consequences, potential harm, and other ethical issues of the school-based HPIs need to be exposed. Finally, this review emphasized a strong need to develop a common standard to implement, report, and evaluate school-based HPIs; the mixed methods evaluation protocol is highly recommended to enhance the scope and rigor of the intervention [93].

However, based on the implemented research process, it is justified to suggest that this study is valid and motivates schoolteachers, health educators, and researchers to collaborate to find more evidence and reliable scientific knowledge on HPIs in school settings. Additionally, the study findings contribute to the field of mixed methods research. This review demonstrates an innovative approach to integrating the qualitative and quantitative methodology through all stages of the literature review, including quantitative and qualitative data collection, analysis, and final inferences. Moreover, mixed methods’ findings offered several additional opportunities for data analyses; e.g., ethical dimensions will be addressed in future studies.

## 5. Conclusions

As a final point, three essential elements of effective school-based HPIs need to be exposed based on the findings of this mixed methods literature review study: (1) multiple target groups; (2) multiple providers, including experts; and (3) a manageable research design with wisely planned follow-ups. The amount of substantial evidence of the effectiveness of interventions in the school setting is dispersed and deficient. Teachers, health professionals, principals, and the academic community are in a central position to support, conduct, and evaluate HPIs targeting primary school pupils’ health. Multiple levels should be taken into account when implementing health-promoting actions, from school policy to an individual level, with strong consideration of the best available evidence. Moreover, ethical discussions regarding health promotion at schools, both in educational and clinical settings, are motivated by scattered evidence. Attention must be given to the quality of interventions’ designs, as well as the relevance and sustainable effects on children’s health. More efforts related to HPIs aimed at mental health are recommended.

## Figures and Tables

**Table 1:** Codes and categories of the structural elements of the school-based HPIs.

Structural Elements of HPI	Codes	Categories	Type of New Variable	Analysis
Health area of intervention	Infection control, physical activity, physical fitness, nutrition, substance abuse, obesity prevention, sleep health, oral health, prevention of anemia, blood pressure, cardiovascular disease prevention, skeletal growth, osteoporosis prevention, spine care, prevention of falls and injuries, healthy lifestyle; Stress management, depression, anxiety, hyperactivity, mindfulness, body image.	Physical health Mental health	Categorical	Thematic analysis
Research design	RCT, stratified randomized trial; Nonrandomized controlled trial, cluster randomized quasi-experiment, nonrandomized pretest–post-test study, prospective controlled study, longitudinal study, prospective longitudinal study, cohort study, prospective cohort study, cross-sectional survey, participatory research approach.	RCT Other designs (quasi-experimental, pretest–post-test, controlled interventions)	Categorical	Thematic analysis
Region of study	Switzerland, Belgium, the United Kingdom, Germany, Sweden, Finland, France, Denmark, the Netherlands, Czech Republic, Italy, Cyprus; Canada, the USA; China, India, Pakistan; Australia; Peru, Bahamas; Kenya.	Europe North America Asia Australia South America Africa	Categorical	Thematic analysis
Target group	Children; Children + one additional group: family, school, teacher, community, peers.	Children Multiple target groups	Categorical	Thematic analysis
Intervention provider	School policy/regulation/curriculum change or improvement; Experts—trained teachers for HPI implementation, trained students, external experts (physiotherapist, registered nurse, school nurse, physician, researcher, dietitian, kinesiologist, psychologist); Multiple providers (≥2 from above).	School policy/curriculum change Experts Multiple providers	Categorical	Thematic analysis
Intervention effectiveness	1—Reverse effect; 2—No effect; 3—Partial/moderate effect; 4—Positive effect; 5—Strong positive effect.	Reverse or no effect Partial effect Positive effect	Numerical, interval	Quantification

**Table 2:** Description of the school-based HPIs included in the review.

Characteristics	Category	Total Interventions	% of Total
**Intervention characteristics**			
Area of intervention	Physical health	40	81.6
	Mental health	9	18.4
Target groups	Children target group	34	69.4
	Multiple target groups	15	30.6
Intervention provider	School policy/curriculum change	23	46.9
	Experts—trained teachers	13	26.5
	External experts	4	8.2
	Multiple providers	9	18.4
Intervention duration (months)	≤2 months	13	26.5
	2.1–12 months	23	47.0
	>12 months	13	26.5
Number of follow-ups	One follow-up	28	57.2
	Two follow-ups	13	26.5
	>2 follow-ups	8	16.3
The first follow-up (months after intervention)	≤3 months	28	57.1
	3.1–6 months	6	12.3
	>6 months	15	30.6
The last follow-up (months after intervention)	≤6 months	20	40.8
	6.1 months–1 year	14	28.6
	>1 year	15	30.6
**Sample characteristics**			
Sample size at the baseline	≤200	9	18.4
	201–600	13	26.5
	601–1199	13	26.5
	≥1200	14	28.6
Age of participants at the intervention baseline	≤8 years	15	30.6
	9–10 years	22	44.9
	≥11 years	12	24.5
**Study characteristics**			
Research design	RCT	30	61.2
	Other designs	19	38.8
Regions	Europe	27	55.1
	North America	8	16.3
	Asia	6	12.3
	Australia	5	10.2
	South America	2	4.1
	Africa	1	2.0

**Table 3:** Hierarchical cluster analysis (Ward’s method) with a three-group solution.

Characteristics	Group 1 Extensive and Long-Term HPI (*n* = 15)		Group 2 School Policy-Changing HPI (*n* = 24)		Group 3 Highly Effective HPI (*n* = 10)		F (*p*)/ χ^2^ (*p*) ^+^
	*n* (%)	M (SD)	*n* (%)	M (SD)	*n* (%)	M (SD)	
Research design—RCT	11 (73.3)		13 (54.2)		6 (60.0)	11 (73.3)	1.036 (0.363) ^+^
Sample size at baseline		1801.93 (2446.24)		901.67 (916.19)		634.70 (692.92)	2.243 (0.118)
Age of participants at baseline		9.30 (1.87)		9.82 (1.47)		9.99 (0.76)	0.798 (0.456)
Intervention duration (months)		25.37 (16.87)		7.53 (7.06)		4.54 (3.53)	16.276 (0.000)
Number of follow-ups		2.40 (1.72)		1.38 (0.58)		1.60 (0.70)	4.257 (0.020)
The first follow-up (months after intervention completion)		18.28 (15.20)		1.83 (2.64)		5.15 (4.26)	16.605 (0.000)
The last follow-up (months after intervention completion)		38.00 (20.09)		4.75 (6.28)		8.20 (4.57)	37.174 (0.000)
Area of intervention—physical health	12 (80.0)		20 (83.3)		8 (80.0)		0.091 (0.956) ^+^
Changing school policy, curriculum	9 (39.1)		13 (56.5)		1 (4.4)		7.009 (0.030) ^+^
Experts and multiple providers ^1^	6 (40.0)		11 (45.8)		9 (90.0)		7.009 (0.030) ^+^
Multiple target groups	5 (33.3)		0 (0.0)		10 (100)		33.307 (0.000) ^+^
Effectiveness—positive and strong positive effects, average effect ^2^ (Total sample: M = 3.39, SD = 1.08).	7 (46.6)	3.27 (1.22)	9 (37.5)	3.08 (0.93)	9 (90.0)	4.30 (0.68)	5.519 (0.007)

^1^ Multiple providers were represented by schoolteachers, specially trained for intervention, or/and by external experts; ^2^ Effectiveness of HPI was coded and quantified into a 5-point interval scale: 1—reverse effect (less as at baseline), 2—no effect, 3—partial/moderate effect, 4—positive effect, 5—strong positive effect remaining at follow-up. The sum of positive and strong positive effective interventions and average effectiveness on the 5-point scale were calculated for each cluster group; F (*p*)—one-way analysis of variance; χ^2^—chi-square test (marked with a cross (+)).

## Data Availability

The dataset related to this manuscript can be made available upon reasonable request.
